# Mortality in severely injured elderly patients: a retrospective analysis of a German level 1 trauma center (2002–2011)

**DOI:** 10.1186/s13049-014-0045-3

**Published:** 2014-08-08

**Authors:** Carsten Schoeneberg, Thomas Probst, Marc Schilling, Alexander Wegner, Bjoern Hussmann, Sven Lendemans

**Affiliations:** 1 Department of Trauma Surgery, University Hospital Essen, University Duisburg-Essen, Hufelandstraße 55, Essen 45147, Germany; 2 Department of Trauma, Orthopedic, and Hand Surgery, Municial Hospital Neuss, Lukashospital GmbH, Preußenstraße 84, Neuss 41464, Germany

**Keywords:** Trauma, Mortality rate, Elderly patients, Severely injured

## Abstract

**Background:**

Demographic change is expected to result in an increase in cases of severely injured elderly patients. To determine special considerations in treatment and outcome, patients aged 75 years and older were studied.

**Methods:**

All patients in the included age group with an Injury Severity Score (ISS) ≥ 16 upon primary admission to hospital between July 2002 and December 2011 were included in this mortality analysis. The data used for this study was gained partly from data submitted to the German Trauma Register and partly from patients’ hospital records. A comparison between survivors and decedents was performed, as well as age-adjusted and ISS-adjusted analyses. The odds ratio and relative risk were used to determine predictors for mortality.

**Results:**

One-hundred eight patients met the inclusion criteria. The overall mortality proportion was 57.4%. The decedents were more severely injured (ISS 26 vs. 20, p < 0.001) and suffered more severe head traumas (GCS 4 vs. 12, p < 0.001; AIS head 5 vs. 4, p = 0.006). No differences were found in vital parameters measured at the accident scene or trauma room. Decedents had deranged coagulation with a prolonged PTT (41.1 sec vs. 27.6 sec, p = 0.008) and reduced prothrombin ratio (66.5% vs. 82.8%, p = 0.016).

Only 17.1% of patients presenting an ISS > 25 survived, suggesting that an injury of such severity is hardly survivable in the subject age group.

Predictors for mortality were: ISS > 25, GCS < 9, PTT > 32.4 seconds, prothrombin ratio < 70%, AIS head > 3, and Hb < 12 g/dl.

**Conclusions:**

The treatment of severely injured elderly patients is challenging. The most common cause of accident is falling from less than 3 m with head injuries being determinant. We identified deranged coagulopathy as an important predictor for mortality, suggesting rapid normalization of coagulation might be a key to reducing mortality.

## Background

Coping with an increasingly aged population is a challenge for healthcare providers all over the world. In 2011, 21% of the German population was aged more than 65 years, compared to 15% in 1990. No other country in the European Union has such a high rate of elderly [1]. This demographic trend is not only a challenge for internists but for all physicians involved in the treatment of elderly trauma patients.

In an American study, the mortality rate of elderly trauma patients increased 3- to 5-fold after adjusting for injury severity [2]. Age is also described as a risk factor for mortality after trauma [3]. Shifflette et al. suggested that all patients aged more than 60 years with multiple injuries and/or significant mechanisms of injury should be transferred to a level 1 trauma center. They found a three-fold increase in morbidity and a five-fold increase in mortality in elderly patients with an ISS between 0 and 15 [4].

Elderly trauma patients have been found to require a greater amount of hospital resources compared to younger patients [5],[6]. Those with an ISS > 30 required less time spend in the intensive care unit (ICU) as a result of increased mortality [5].

In an Australian study, the rate of severe trauma to older patients increased by nearly 5% per year, with one third of all trauma admissions being elderly patients [7].

Elderly patients often present comorbid conditions, concomitant medication (especially anticoagulation medication), and lower physiologic reserve compared to younger trauma patients. These factors reduce their ability to respond to aggressive trauma resuscitation, and injury impact is greater compared to younger patients [8]. Concomitant medication has been shown to frequently alter the hemodynamic response to shock [9], and, because of the absence of hypotension and tachycardia, injury severity and response to resuscitation could be underestimated [10],[11].

To anticipate predicted demographic changes and to address an underrepresentation of the oldest patients in the literature, the aim of this study was to determine special considerations in treatment and outcome in patients aged greater than 75 years.

## Material and methods

### General information

This study analyzed the data of a level 1 university-based trauma center in Germany. Serving the catchment area of the Ruhr district with approximately 5.1 million habitants, it is one of the largest trauma centers in Germany. There are four level 1 trauma centers in the Ruhr district and the emergency medical system is mostly ground based, though air transportation via helicopter is available as well. For our hospital, the rate of helicopter ambulance is approximately 11%.

The data used in this study was collected prospectively for the national trauma registry, called the Trauma Registry of the German Society for Trauma Surgery (DGU). The data from the Trauma Registry of the DGU has received full approval from the Ethics Committee of the University of Witten/Herdecke in Cologne, Germany. Because the trauma registry of the DGU is an anonymous register, the Institution Review Board waived the need for patient consent. Additionally, the patients’ clinical records were analyzed. For this analysis we received full approval from the Ethics Committee of the medical faculty of the University Duisburg-Essen in Essen, Germany.

### Patients

Inclusion and exclusion criteria are shown in Table 1.

**Table 1:** Inclusion and exclusion criteria applied in this study

**Inclusion criteria**	**Exclusion criteria**
Primary admission to the hospital	Transfer from other hospital
Activation of the trauma team	No activation of the trauma team
ISS ≥ 16	ISS < 16
≥ 76 years of age	< 76 years of age
Admission occurred between July 2002 and December 2011	

Scales, general patient information, laboratory test values, and intervention data were collected for each patient as follows. Scales: Injury Severity Score (ISS) [12]; Abbreviated Injury Scale (AIS); New ISS [13]; Glasgow Coma Scale (GCS) [14]; Revised Trauma Score (RTS) [15]; Revised Injury Severity Classification (RISC) [16]; and Trauma and Injury Severity Score (TRISS).

General patient information: Age; sex; ASA score; systolic blood pressure at the accident scene and at admission; heart rate at the accident scene and at admission; oxygen saturation at the accident scene and at admission; length of ICU stay; length of hospital stay; count of performed surgeries; administered fluid volume; proportion of multi-organ failure (MOF); proportion of sepsis; and type of injury (penetrating vs. blunt).

Laboratory test values: First hemoglobin (Hb) value; initial number of platelets; partial Thromboplastin time (PTT); Prothrombin time; base excess; and lactate.

Length of analyzed periods: Time from admission to cranial computed tomography (CCT); time from admission to whole-body CT; time in trauma room; time from admission to operating room; and preclinical rescue time (time from arriving at the accident scene to admission in hospital).

Interventions: Intubation, resuscitation, and thoracic drainage by emergency physician at the accident scene and intubation, cardio-pulmonary resuscitation, and thoracic drainage in trauma room.

To allow age-adjusted analysis, the patients were divided into age groups as follows: 76–80 years, 81–85, 86–90, and > 90 years of age.

Similarly, patients were grouped by ISS: ISS 16–25, 26–35, 36–45, and > 45.

To determine the difference between decedents and survivors, odds ratios and relative risks with 95% confidence intervals (CI) were used to determine predictors for mortality. The cutoffs for laboratory tests were set according standard values (Table 2).

**Table 2:** Standard values for laboratory tests

**Laboratory test**	**Standard value**
Hemoglobin	12.0 – 15.2 g/dl
Platelets	180 – 380 gpt/l
Partial Thromboplastin time (PTT)	24.4 – 32.4 sec
Prothrombin ratio	70 – 130%
Base excess	-2.0 – 2.0 mmol/l
Lactate	0.5 – 2.2 mmol/l

### Statistics

Data were analyzed using the Statistical Package for the Social Sciences (SPSS21; IBM Company; Chicago, IL, USA). Incidences are represented as percentages. Measured values are represented as means and 95% CI for continuous variables, and for categorical variables as medians and interquartile ranges (IQR). Differences were evaluated using the Chi-squared test for categorical variables and the *t*-test for continuous variables. When performing the *t*-test, Levene’s test was also performed. In cases of variance heterogeneity, the Welch-test was used instead of the *t*-test. Normal distribution was tested using the Kolmogorov-Smirnov-test. When an obvious deviation from normality was detected, continuous variables were tested with a non-parametric rank test (Mann–Whitney test). Differences were considered statistically significant when p < 0.05.

## Results

### General results

In the observation period, 2,304 patients were admitted to the trauma room. Of these, 258 patients were aged more than 75 years. A total of 108 patients met the inclusion criteria, of which 38.3% were male.

**Figure 1:**
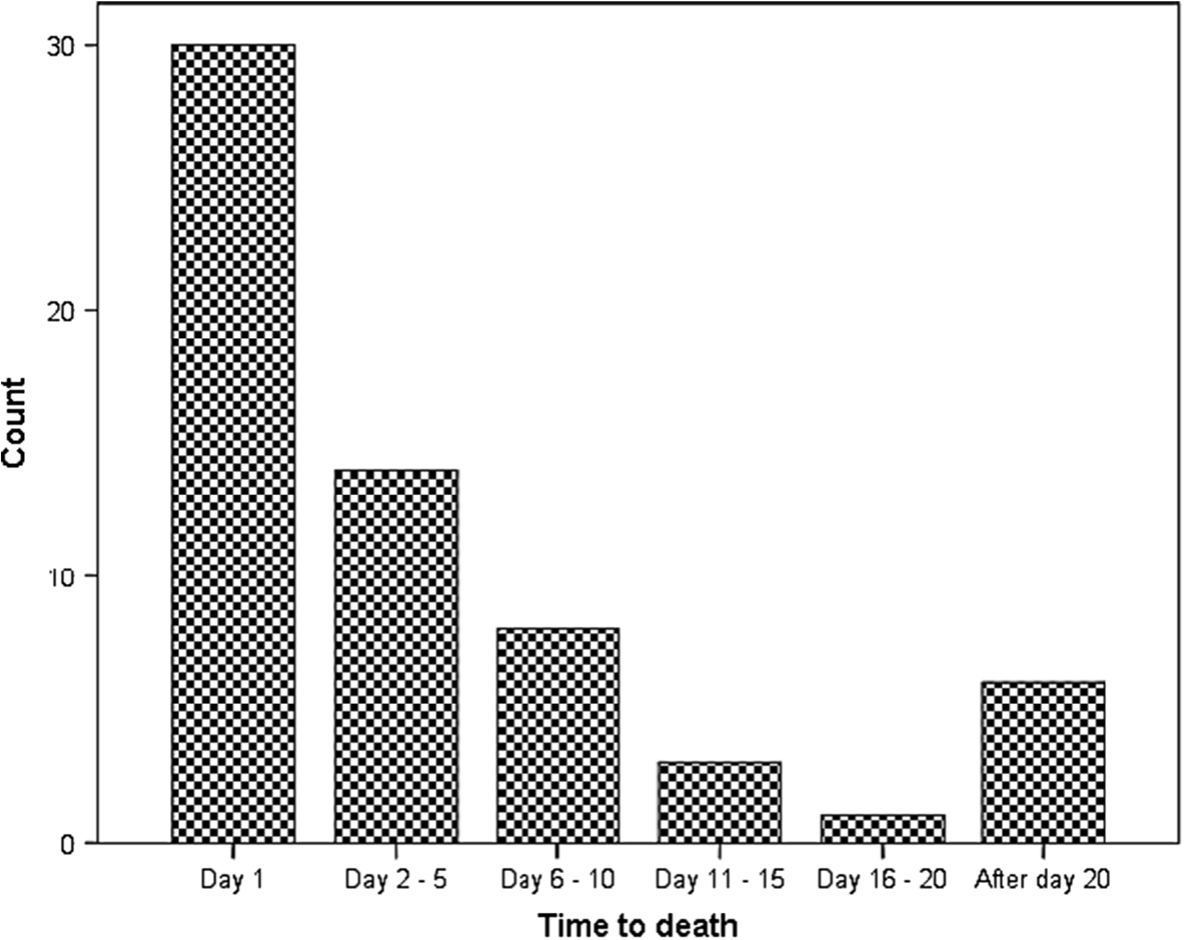
Time to death in severely injured elderly patients.

The median GCS was 6 (3–13), the median ISS was 25 (20–29), the median AIS head was 5 (4–5), and the mean age was 82.2 years. 62 patients died after trauma, resulting in a mortality proportion of 57.4%. The expected mortality proportion, demonstrated by the RISC, was 54.6%. Most of the patients (29; 46.8%) died within the first 24 hours (Figure 1). In addition, 15 (24.2%) died between days 2 and 5, 8 (12.9%) between days 6 and 10, and 10 (16.1%) after day 10. The in-hospital mortality was analyzed.

**Figure 2:**
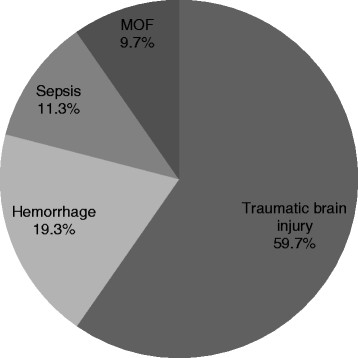
Cause of death in severely injured elderly patients.

The primary cause of death was severe head injury, claiming 37 (59.7%) patients by traumatic brain injury. In addition, 12 (29.4%) patients died as a result of hemorrhage, 7 (11.3%) as a result of sepsis, and 6 (9.7%) as a result of MOF (Figure 2).

### Comparisons between decedents and survivors

Differences between survivors and decedents are shown in Table 3.

**Table 3:** Differences between survivors and decedents

	**Decedents**	**Survivors**	**p value**
**GCS**	4 (3 - 9)	12 (6 - 15)	< 0.001*
**RTS**	5.2 (4.5 – 5.9)	6.6 (5.8 – 7.3)	= 0.003*
**ISS**	26 (25 - 33)	20 (17 - 25)	< 0.001*
**TRISS**	0.4 (0.3 – 0.6)	0.8 (CI 0.7 – 0.9)	< 0.001*
**RISC**	69.6 (63.4 – 75.9)	34.5 (27.0 – 42.1)	< 0.001*
**AIS Head**	5 (4 - 5)	4 (3 - 4)	= 0.006*
**ASA**	2 (2 - 2)	2 (2 - 2)	= 0.325
**Age**	81.9 (80.7 – 83.2)	82.6 (81.1 – 84.2)	= 0.511
**SBR AS**	140 (100 - 160)	140 (120 - 160)	= 0.381
**Heart rate AS**	88 (80 - 100)	88 (80 - 100)	= 0.839
**Oxygen saturation AS (%)**	90 (80 - 96)	93 (89 - 96)	= 0.117
**SBP TR**	125 (93 - 152)	142 (113 - 161)	= 0.070
**Heart rate TR**	87 (70 - 107)	87 (76 - 100)	= 0.804
**Oxygen saturation TR (%)**	99 (96 - 100)	98 (95 - 100)	= 0.396
**Hb (g/dl)**	10.5 (9.9 – 11.1)	11.6 (11.0 – 12.3)	= 0.004*
**PTT (sec.)**	41.1 (30.0 – 52.3)	27.6 (24.4 – 30.8)	= 0.008*
**Prothrombin ratio (%)**	66.5 (57.3 – 75.6)	82.8 (73.9 – 91.8)	= 0.016*
**Base excess**	-4.6 (-6.3 - -3.0)	-2.7 (-4.5 – 1.0)	= 0.129
**Lactate (mmol/l)**	2.9 (1.8 – 3.9)	1.6 (1.1 – 2.0)	= 0.043*
**ICU stay (days)**	6.5 (3.6 – 9.5)	15.6 (10.0 – 21.1)	< 0.001*
**Hospital stay (days)**	7.5 (4.4 – 10.6)	20.2 (14.9 – 25.5)	< 0.001*
**Pre-hospital volume (ml)**	875.0 (698.1 – 1051.9)	869.6 (665.1 – 1074.1)	= 0.982
**TR volume (ml)**	1506.6 (1153.2 – 1860.0)	1283.4 (876.9 – 1690.0)	= 0.252
**Total volume (ml)**	2439.7 (1966.4 – 2913.1)	2215.5 (1613.8 – 2817,2)	= 0.321
**Time in TR (min)**	51.9 (45.8 – 58.0)	54.7 (46.9 – 62.4)	= 0.710
**Gender Male**	37.1%	40%	= 0.760
**Rate of whole-body CT**	53.6%	56.8%	= 0.763
**MOF**	51%	23.3%	= 0.006*
**Sepsis**	15.8%	25.0%	= 0.249
**Intubation at AS**	77.4%	40%	< 0.001*
**Intubation in TR**	40.7%	43.5%	= 0.773

Categorical variables are presented as median with the interquartile range in parentheses, continuous variables as means with the 95% confidence interval in parentheses, and incidences as percentages; *significant differences.

**Figure 3:**
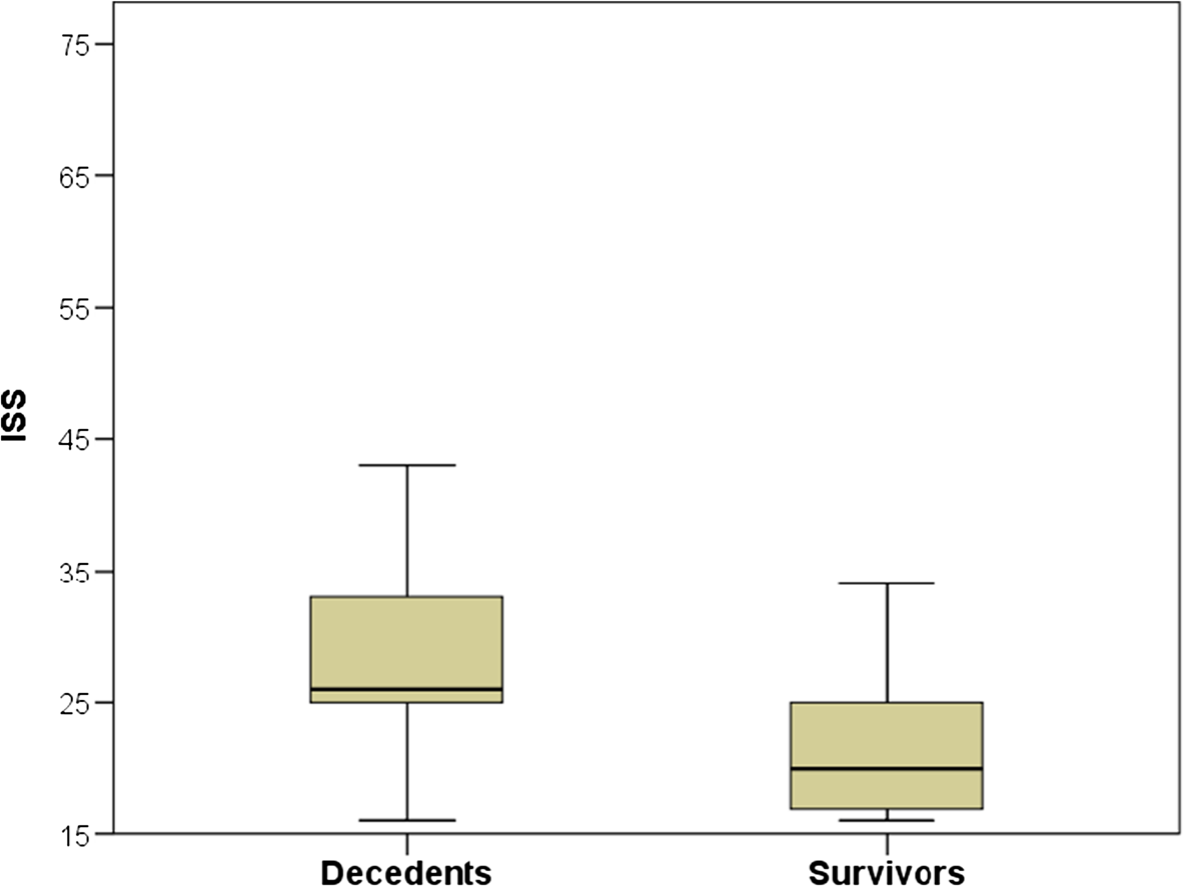
Box-plot diagram of the ISS divided into decedents and survivors.

The decedents were more severely injured, having a higher ISS (26 vs. 20, p < 0.001) (Figure 3) compared to survivors. Decedents suffered more severe head trauma, evidenced by a lower GCS (4 vs. 12, p < 0.001), and the higher AIS head score (5 vs. 4, p = 0.006). No differences were found in other the AIS scores.

There was no statistical difference in the ASA score, suggesting the two groups were similar regarding comorbid conditions. There were also no differences in the physiologic parameters, such as systolic blood pressure, heart rate, or oxygen saturation, whether at the accident scene or in the trauma room.

The first hemoglobin count was different between the groups, with lower values in the decedents (10.5 vs. 11.6 g/dl, p = 0.004). The coagulation values were different as well. The PTT was extended to 41.1 seconds in decedents compared to 27.6 seconds in survivors (p = 0.008). Prothrombin ratio was lower in the decedents (66.5% vs. 82.8%, p = 0.016). No difference was found in platelet counts.

Although the base excess was not different between the two groups, the lactate value was higher in the decedents than in survivors (2.9 vs. 1.6 mmol/l, p = 0.043).

The lengths of stay in the ICU and in hospital were shorter in the decedents. All other investigated time periods were not different.

**Figure 4:**
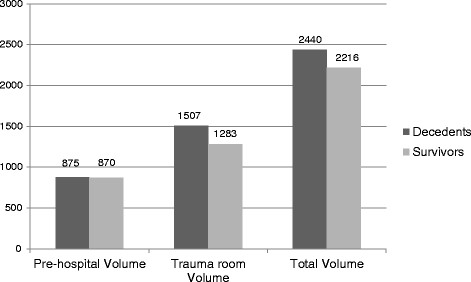
Distribution of the infused fluid volume divided by decedents and survivors.

No differences were found in the fluid volumes the patients received. In total, decedents received 2439.7 ml and the survivors received 2215.5 ml (p = 0.321). 52 patients received more than 1500 ml fluid volume. Of these, 23 presented normal physiologic parameters at the accident scene and in the trauma room (systolic blood pressure ≥ 120 mm Hg, pulse rate < 100 bpm). The blood pressure cutoff of 120 mm Hg was used, because no emergency physician might consider such a blood pressure as hemorrhage-inducted hypotension. The distribution of infused fluid volumes is presented in Figure 4.

Decedents suffered from MOF more often (51% vs. 23.3%). Analysis by organ system resulted in a higher proportion of failure in decedents in almost every system, respiratory (56.1% vs. 23.3%; p = 0.001), central nervous system (86.4% vs. 43.5%; p < 0.001), heart and circulatory system (71.2% vs. 18.6%; p < 0.001), and renal system (22% vs. 4.8%; p = 0.018). Failure rate in only the hepatic system was not different between the groups.

The results of determining odds ratios and relative risks are shown in Table 4. Six predictors for mortality in elderly trauma patients were identified: ISS > 25, GCS < 9, PTT > 32.4 seconds, prothrombin ratio < 70%, AIS head > 3, and Hb < 12 g/dl. The strongest predictor for mortality was ISS > 25, with a 7-fold higher risk for a fatal course.

**Table 4:** Odds ratios and relative risk of the differences between decedents and survivors

**Variable**	**Odds ratio (95% CI)**	**Relative risk (95% CI)**	**p value**
**ISS > 25**	6.77 (2.62 – 17.45)	3.60 (1.76 – 7.40)	< 0.001
**GCS < 9**	4.53 (1.97 – 10.40)	1.98 (1.30 – 3.02)	< 0.001
**AIS Head > 3**	2.77 (1.12 – 6.88)	1.29 (1.01 – 1.63)	= 0.025
**Hb < 12 g/dl**	2.65 (1.19 – 5.91)	1.45 (1.05 – 2.01)	= 0.016
**PTT > 32.4 sec**	3.62 (1.13 – 11.57)	2.67 (1.06 – 6.73)	= 0.025
**Prothrombin ratio < 70%**	2.39 (1.02 – 5.55)	1.69 (1.00 – 2.88)	= 0.042
**Lactate > 2.2 mmol/l**	2.07 (0.59 – 7.29)	1.65 (0.68 – 4.03)	= 0.253

ISS, Injury Severity Score; GCS, Glasgow Coma Scale; AIS Abbreviated Injury Scale; Hb, Hemoglobin; PTT, Partial Thromboplastin time.

The most common cause of trauma was fall from a height less than 3 m (for decedents and survivors), followed by accidents as a pedestrian. No differences occurred between decedents and survivors according the cause of trauma (Table 5).

**Table 5:** Cause of injury

**Cause of injury**	**Total**	**Decedents**	**Survivors**	**p value**
**Traffic accident, car**	3.7%	4.8%	2.2%	= 0.478
**Traffic accident, motorcycle**	1.9%	1.6%	2.2%	= 0.831
**Traffic accident, bicycle**	3.7%	3.2%	4.4%	= 0.760
**Traffic accident, pedestrian**	22.2%	21%	23.8%	= 0.716
**Fall > 3m**	10.2%	12.9%	6.5%	= 0.557
**Fall < 3m**	51.9%	50%	54.4%	= 0.618
**Others**	6.4%	6.5%	6.5%	= 0.577

The total number of cases and proportion of cases in decedents and survivors are shown.

### Age-adjusted analysis

**Figure 5:**
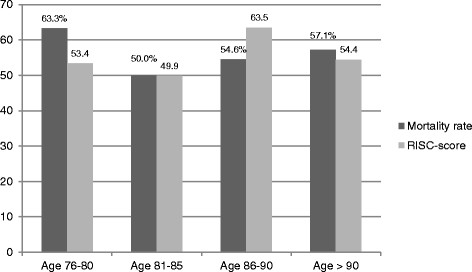
Mortality and RISC-score in the age-adjusted groups.

The mortality proportion was similar in all age-adjusted groups with no great difference to the prognostic mortality proportion, as reported by the RISC-score (Figure 5). In all age-groups, the observed and expected mortality rate was 50% or greater.

The preclinical intubation proportion was different between survivors and decedents. Most decedents were intubated at the accident scene. This difference was found in all age-groups.

Looking at the group of patients aged less than 86 years, survivors had a significantly higher GCS. Only in the youngest group (76–80 years of age) did the decedents suffer more MOF than the survivors. In older patients, no difference in MOF and sepsis proportion occurred between decedents and survivors. There were no differences in AIS scores between groups (Table 6).

**Table 6:** Significant differences in decedents and survivors divided by age

**Age**		**Decedents**	**Survivors**	**p value**
**76-80**	Patients	31	18	
	GCS	6 (3 - 12)	14 (9 - 15)	= 0.004
	ISS	26 (25 - 33)	20 (17 - 25)	< 0.001
	TRISS	0.5 (0.4 – 0.6)	0.8 (0.6 – 1.0)	= 0.001
	RISC	68.6 (59.2 – 78.1)	27.9 (17.8 – 38.0)	< 0.001
	Intubation at AS	71%	35.3%	= 0.017
	Thoracic drainage in TR	21.4%	0%	= 0.035
	MOF	56%	25%	=0.050
	Mortality proportion	63.3%		
	RISC overall	53.4		
**81-85**	Patients	15	15	
	GCS	3 (3 - 8)	13 (6 - 15)	= 0.002
	RTS	4.5 (2.8 – 6.1)	7.1 (6.4 – 7.8)	= 0.003
	ISS	26 (25 - 36)	19 (17 - 25)	= 0.007
	TRISS	0.4 (0.2 – 0.6)	0.9 (0.8 – 1.0)	< 0.001
	RISC	69.3 (54.2 – 84.4)	30.5 (15.1 – 45.8)	< 0.001
	Heart rate at AS	100 (90 - 120)	80 (76 - 84)	= 0.015
	Heart rate in TR	112 (80 - 123)	82 (76 - 100)	= 0.037
	PTT (sec)	56.4 (13.0 – 99.9)	29.6 (22.9 – 36.2)	= 0.008
	Intubation at AS	73.3%	33.3%	= 0.028
	Mortality proportion	50.0%		
	RISC overall	49.9		
**86-90**	Patients	12	10	
	ISS	26 (25 - 29)	24 (17 - 25)	= 0.007
	RISC	74.4 (64.5 – 84.4)	50.4 (32.3 – 68.5)	= 0.030
	Intubation at AS	100%	70%	= 0.041
	Mortality proportion	54.6%		
	RISC overall	63.5		
**> 90**	Patients	4	3	
	Intubation at AS	75%	0%	0.047
	Mortality proportion	57.1%		
	RISC overall	54.4		

Categorical variables are presented as median with the interquartile range in parentheses, continuous variables as means with the 95% confidence interval in parentheses, and incidences as percentages; GCS, Glasgow Coma Scale; ISS, Injury Severity Score; TRISS, Trauma and Injury Severity Score; RISC, Revised Injury Severity Classification; AS, Accident scene; TR, Trauma room; MOF, Multi-organ failure; PTT, Partial Thromboplastin time.

### ISS-adjusted analysis

The ISS-adjusted analysis, presented in Table 7, showed differences between decedents and survivors only in those scoring 16–25. Only 10 patients presented an ISS > 45 (Table 7).

**Table 7:** Significant differences between decedents and survivors divided by ISS

**ISS**		**Decedents**	**Survivors**	**p value**
**16-25**	Patients	28	39	
	GCS	5 (3 - 10)	12 (9 - 15)	= 0.002
	RTS	5.4 (4.3 – 6.6)	6.7 (5.9 – 7.4)	= 0.028
	ISS	25 (25 - 25)	18 (17 - 24)	< 0.001
	TRISS	0.5 (0.4 – 0.7)	0.8 (0.7 – 0.9)	= 0,003
	RISC	63.7 (54.5 – 73.0)	31.4 (23.4 – 39.4)	< 0.001
	PTT (sec)	43.6 (24.1 – 63.0)	27.2 (23.7 – 30.8)	= 0.012
	Prothrombin value (%)	59.1 (46.7 – 71.5)	82.9 (72.7 – 93.1)	= 0.004
	AIS head	5 (4 - 5)	4 (3 - 4)	= 0.006
	Intubation at AS	78.6%	36.8%	= 0.001
	Mortality proportion	40%		
	RISC overall	44.9		
**26-35**	Patients	20	3	
	GCS	6 (3 - 8)	14 (13 - 14)	= 0.003
	AIS Thorax	0 (0 - 2)	3 (2 - 3)	= 0.042
	Male	25%	100%	= 0.030
	Mortality proportion	87%		
	RISC overall	62.5		
**36-45**	Patients	5	3	
	Mortality proportion	62.5%		
	RISC overall	64.5		
**> 45**	Patients	9	1	
	Mortality proportion	90%		
	RISC overall	93.8		

Categorical variables are presented as median with the interquartile range in parentheses, continuous variables as means with the 95% confidence interval in parentheses, and incidences as percentages; GCS, Glasgow Coma Scale; RTS, Revised Trauma Score; ISS, Injury Severity Score; TRISS, Trauma and Injury Severity Score; RISC, Revised Injury Severity Classification; PTT, Partial Thromboplastin time; AS, Accident scene; AIS, Abbreviated Injury Scale.

**Figure 6:**
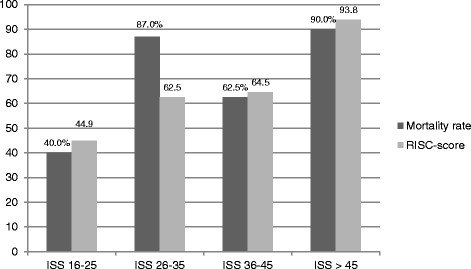
Mortality and RISC-score in the ISS-adjusted groups.

The surviving-proportion was only 17.1% among patients presenting an ISS > 25. The ISS-adjusted mortality proportion and the expected mortality proportion are shown in Figure 6.

In the ISS 16–25 group, decedents suffered more severe head injury than survivors, evidenced by lower GCS and significantly higher AIS score head. Also, decedents had deranged coagulation with significantly lower prothrombin ratio and a prolonged PTT, compared to survivors.

## Discussion

Predicted demographic change will result in trauma physicians being faced with a higher rate of elderly trauma patients. This study focused on severely injured patients aged greater than 75 years.

In this study, the overall mortality proportion was 57.4%. In the same time period, the mortality proportion of all treated severely injured patients in the same hospital was 28.7% [17]. When patients aged more than 75 years were excluded, the mortality proportion was 24.9%. This result in a 2-fold increase of mortality proportion, which is less than previously reported [2].

Similar to Taylor et al. we found greater mortality associated with a higher ISS [5]. Only 17.1% of the patients who presented with an ISS greater than 25 survived. The median ISS of survivors and decedents were 20 and 26, respectively. Richmond et al. reported that when ISS was greater than 25, the odds for a fatal course was raised by a factor of 25 [18]. Our analysis yielded an increase by a factor 7, showing that ISS was the strongest predictor for mortality.

Among AIS scores, we identified the AIS head score as the only one that differed between decedents and survivors. Therefore, it would seem that the decedents suffered more severe head injuries, and that the head injuries are the determinant injuries.

The decedents typically had deranged coagulation when arriving at the trauma room. The mean PTT was prolonged to 41.1 seconds and the mean prothrombin ratio was reduced to 66.5%. One possible explanation is that a severe head injury caused a coagulopathy because of expression of tissue thromboplastin and tissue factor from the injured brain [19]-[21]. Another explanation could be that elderly patients are more likely to be taking anticoagulant medication. However, this could not be confirmed in our data.

In 23 patients, the heart rate and systolic blood pressure were in the physiologic range at the accident scene and in the trauma room; however, they received over 1500 ml fluid volume in total. According to the S3 guideline of the DGU, patients with a normal systolic blood pressure should not receive fluid volume [22]. Also, Ley et al. reported a higher mortality rate in elderly patients who received more than 1500 ml [23]. We did not find this to be the case, because both the decedents and the survivors received more than 2000 ml volume. Therefore, it might be possible that the mortality proportion would have been lower if less volume had been infused.

In a recently published study, Salottolo et al. reported that lactate is a predictor of mortality in elderly patients [24]. In our study, there was a significantly higher lactate value in decedents than survivors, supporting the idea that lactate might be a predictor for mortality. However, the odds ratios showed that lactate was not an independent predictor for mortality. Salottolo used 2.5 mM as the cut-off, while we used the standard value cut-off (Table 2).

Interestingly, only 38.3% of the patients were male. In an analysis of trauma patients without limitations to age, the proportion of male patients is greater than two-thirds. In the patient group this study is based on the proportion was 71.1% [17]. In 2010, the life expectancy in Germany for males was 77.3 years, compared to 82.5 years for females. Therefore, it is to be expected that among people aged more than 75 years, the proportion of females is higher. This might also be an explanation for the relatively low proportion of sepsis in the decedents, because studies investigating gender differences report lower sepsis rates among females than males [25]-[27].

Analyses of the causes of accidents found that most were of low impact, such as a fall from less than 3 m. The second most frequent cause was a traffic accident as a pedestrian. These findings are similar to other recently published studies [7],[28],[29].

When analyzing mortality in subgroups by age, no differences were found between groups, suggesting that age greater than 75 years is not a predictor for mortality.

In our analysis, we identified six predictors for mortality in elderly trauma patients: ISS > 25, GCS < 9, PTT > 32.4 seconds, prothrombin ratio < 70%, AIS head > 3, and Hb < 12 g/dl.

### Limitations

This study is limited because it is a retrospective analysis. Also, as a single-center study, there might be selection for patients treated in our hospital. To confirm these results, a trauma registry analysis with multicenter data should be performed. No statement can be made about patients who died at the accident scene or on the way into hospital because no documentation is available for these patients. This might introduce a bias, although this study investigated only the in-hospital mortality.

## Conclusion

Our findings suggest that the treatment of severely injured elderly patients is a challenging one. Due to predicted demographic changes this group of patients is expected to become increasingly important. The determinant injuries were head injuries and it appears that deranged coagulopathy is an important predictor for mortality. Therefore, rapid normalization of coagulation and, if possible, establishing a medical history of anticoagulant medication use might be important in elderly trauma patients.

## Abbreviations

AIS: Abbreviated injury scale

AS: Accident scene

ASA: American Society of Anesthesiologists

CCT: Cranial computed tomography

CI: Confidence interval

DGU: German Society for Trauma Surgery

GCS: Glasgow coma scale

Hb: Hemoglobin

ICU: Intensive care unit

IQR: Interquartile range

ISS: Injury severity score

MOF: Multi-organ failure

PTT: Partial Thromboplastin time

RISC: Revised injury severity classification

ROC: Receiver operating characteristic

RTS: Revised trauma score

SBP: Systolic blood pressure

SPSS: Statistical Package for the Social Sciences

TR: Trauma room

TRISS: Trauma and injury severity score

vs.: Versus

## Competing interests

The authors declare that they have no competing interests.

## Authors’ contributions

CS and SL designed this study. CS, TP, MS, AW and BH collected and analyzed the data. CS drafted the manuscript, and all authors contributed substantially to its revision. CS takes responsibility for the paper as a whole. All authors read and approved the final manuscript for publication.
